# Pigmented Oral Lesions: A Multicenter Study

**DOI:** 10.1055/s-0041-1735790

**Published:** 2021-11-09

**Authors:** Kittipong Dhanuthai, Natchaya Theungtin, Natnicha Theungtin, Pantira Thep-akrapong, Sompid Kintarak, Poramaporn Klanrit, Nutchapon Chamusri, Kraisorn Sappayatosok

**Affiliations:** 1 Department of Oral Pathology, Faculty of Dentistry, Chulalongkorn University, Bangkok, Thailand; 2 Faculty of Dentistry, Chulalongkorn University, Bangkok, Thailand; 3 Department of Oral Diagnostic Sciences, Faculty of Dentistry, Prince of Songkla University, Songkhla, Thailand; 4 Department of Oral Biomedical Sciences, Faculty of Dentistry, Khon Kaen University, Khon Kaen, Thailand; 5 Department of Oral Biology and Oral Diagnostic Sciences, Chiang Mai University, Chiang Mai, Thailand; 6 Department of Oral Diagnostic Sciences, College of Dental Medicine, Rangsit University, Pathumtani, Thailand

**Keywords:** clinical features, pigmented lesions, prevalence, Thailand

## Abstract

**Objectives** The aim of this study was to determine the prevalence and clinical features of pigmented oral lesions from Thailand.

**Materials and Methods** Biopsy records of the Department of Oral Pathology, Chulalongkorn University, Department of Oral Diagnosis, KhonKaen University, Department of Oral Biology and Oral Diagnostic Sciences, Chiangmai University, Department of Stomatology, Prince of Songkla University, and Rangsit University were reviewed for oral pigmented lesions diagnosed during 1999 to 2019. Demographic data were culled from the biopsy records. Ages of the patients were subdivided into 10-year intervals. Locations of the lesions were classified as gingiva, labial/buccal mucosa, palate, floor of the mouth, tongue, as well as the combination of sites. Data were analyzed by descriptive statistics using SPSS version 20.0.

**Results** Of the 47,175 accessioned cases, 241 cases (0.51%) were diagnosed in the category of pigmented oral lesions. The age of the patients ranged from 1 month to 88 years with the mean ± standard deviation = 38.74 ± 20.96 years. Regarding gender, 172 patients (71.37%) with pigmented lesions were females, while 69 patients (28.63%) were males. The female-to-male ratio was 2.49:1. The majority of the pigmented lesions were encountered at the gingiva (29.88%) followed by labial/buccal mucosa (26.97%), palate (14.94%), lip (10.79%), alveolar mucosa (9.54%), and others (7.88%), respectively. The three most common pigmented oral lesions in the present study were nevus (39.83%), followed by melanotic macule (28.63%) and amalgam tattoo (17.43%), respectively.

**Conclusions** The most common pigmented oral lesion in the present study is nevus. Demographic data of the patients in the present study are in accordance with previous studies with minor differences. Even though pigmented lesions of the oral cavity constitute a small portion of the oral pathology biopsies, accurate diagnosis is important since there is an overlap in clinical appearance of benign pigmented lesions and melanoma.

## Introduction

The color of the oral mucosa varies from pink to white to red-purple in persons with pale-skinned persons to brown to black in dark-skinned persons depending on the degree of keratinization, thickness, vascularization, the number of melanocyte, melanocytic activities as well as the type of submucosal tissue. 1 2 Oral pigmentation can be the result of physiologic or pathologic condition. Pathologic conditions can be classified as endogenous or exogenous depending on the cause. Endogenous pigmentation ranges from melanotic macule, melanoacanthoma, postinflammatory pigmentation, so-called smoker's melanosis, melanocytic nevus, Peutz-Jeghers syndrome, Addison's disease, melanosis associated with systemic diseases to melanoma. Exogenous pigmentation encompasses heavy metal pigmentation, and foreign body such as amalgam/graphite tattoo. Drug-induced melanosis can be classified as exogenous pigmentation from the direct deposit on the tissue or endogenous pigmentation from the induced increased melanin production. 2 3 Oral pigmentation can also be classified according to the clinical presentation as focal or solitary pigmentation versus multifocal or diffuse pigmentation. 4

The studies on oral pigmentation as a whole are rather limited. Most of the previous studies focused on a single entity. 5 6 To the best of our knowledge, there have been only two studies 3 4 on oral pigmentation from the oral pathology diagnostic centers. The aims of this study were to determine the prevalence and the clinicopathologic features of the patients with oral pigmentation from Thailand.

## Materials and Methods

Biopsy records of the Department of Oral Pathology; Chulalongkorn University, Department of Oral Diagnosis; KhonKaen University, Department of Oral Biology and Oral Diagnostic Sciences; Chiangmai University, Department of Stomatology; Prince of Songkhla University and Department of Oral Diagnostic Sciences; Rangsit University were reviewed for oral pigmented lesions diagnosed during 1999 to 2019. Demographic data were culled from the biopsy records. Ages of the patients were subdivided into 10-year intervals. Locations of the lesions were classified as gingiva, labial/buccal mucosa, palate, floor of the mouth, tongue, as well as the combination of sites. Lip was defined as the vermilion part of the lip (lip body). Data were analyzed by descriptive statistics using SPSS version 20.0.

## Results

Of the 47,175 accessioned cases, 241 cases (0.51%) were diagnosed as pigmented oral lesions. The prevalence ranged from 0.32% from Chulalongkorn University in Central Thailand to 1.35% from KhonKaen University in the Northeast of Thailand. The age of the patients ranged from 1 month to 88 years with the mean ± standard deviation = 38.74 ± 20.96 years. The majorities of the patients (62.24%) fell in the third to the sixth decades of life. Seventeen cases (7.05%) were discovered in children aged 16 and below. Twenty-eight cases (11.62%) were found in the elderly aged 65 and above. Mean age of the pediatric patients was 11.76, while that of the elderly patients was 71.93 years.

Regarding gender, 172 patients (71.37%) with pigmented lesions were females, while 69 patients (28.63%) with pigmented lesions were males. The female-to-male ratio was 2.49:1. All participating institutions demonstrated a higher number of female patients than male patients. Both the pediatric and the elderly groups also elicited a female predilection. All pathologic entities demonstrated a female preponderance.

The majority of the pigmented lesions were encountered at the gingiva (29.88%) followed by labial/buccal mucosa (26.97%), palate (14.94%), lip (10.79%), and alveolar mucosa (9.54%), and others (7.88%), respectively. In the pediatric patients, the most common sites for pigmented oral lesions were labial/buccal mucosa (47.06%) followed by lip (23.53%). In the geriatric patients, the most common sites for pigmented oral lesions were labial/buccal mucosa and gingiva (25.00% each) followed by palate (21.43%).

**Fig. 1:**
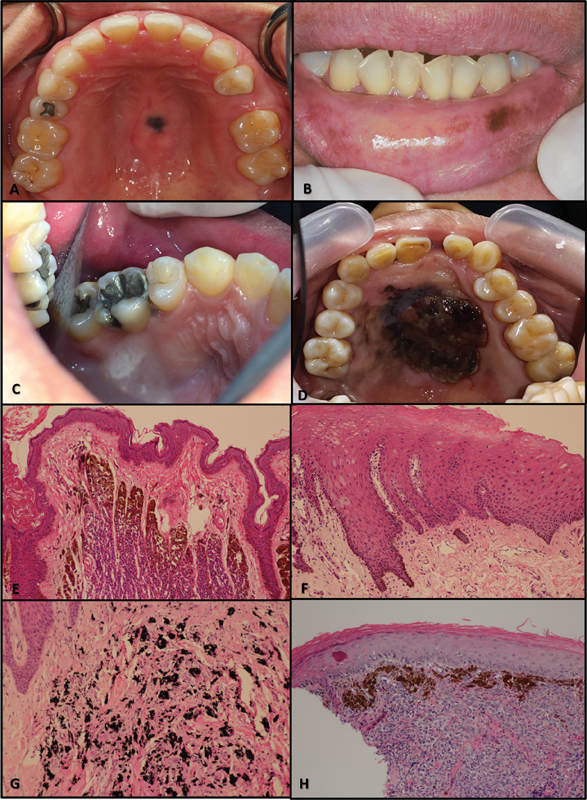
Clinical features of oral pigmented lesions: ( **A** ) Nevus, ( **B** ) melanotic macule, ( **C** ) amalgam tattoo, and ( **D** ) melanoma. Histopathologic features of oral pigmented lesions: ( **E** ) Nevus, ( **F** ) melanotic macule, ( **G** ) amalgam tattoo, and ( **H** ) melanoma. (Hematoxylin and eosin stain, original magnification 100X). Clinical picture of melanoma courtesy of Lieutenant Poon Notklao, Queen Sirikit Naval Hospital, Thailand.

Nevus was the most prevalent pigmented oral lesion (39.83%) followed by melanotic macule (28.63%) and amalgam tattoo (17.43%), respectively. The histopathological features oral pigmented lesions are shown in Fig. 1 . Within the nevus group, intramucosal nevus was the most common lesion accounting for 66.67% of all nevi, followed by compound nevus (25.00% of all nevi) and blue nevus (8.33% of all nevi), respectively. Table 1 shows the clinicopathologic features of the patients with pigmented oral lesions in the study. In the pediatric patients aged 16 years and younger, the most common pigmented lesion was compound nevus (52.94% of the pediatric patients), followed by intramucosal nevus (23.53% of the pediatric patients), and melanotic macule (11.76% of the pediatric patients), respectively. In the elderly patients aged 65 years and older, the most common pigmented lesion was melanotic macule (35.71% of the elderly patients), followed by amalgam tattoo and melanoma (25.00% of the elderly patients each).

**Table 1:** Clinicopathologic features of the patients with pigmented oral lesions

Diagnosis	Number (%)	Mean age ± SD	F:M ratio	Most common location
Nevus	96 (39.83%)	29.80 ± 17.91	2.20:1	Labial/buccal mucosa (41.67%)
–Intramucosal nevus	64 (26.56%)	30.81 ± 17.58	2.76:1	Labial/buccal mucosa (51.56%)
–Compound nevus	24 (9.96%)	26.33 ± 19.87	1.66:1	Lip (20.83%)
–Blue nevus	8 (3.31%)	32.13 ± 14.78	1.66:1	Palate (50%)
Melanotic macule	69 (28.63%)	40.83 ± 20.42	3.06:1	Gingiva (33.33%)
Amalgam tattoo	42 (17.43%)	45.02 ± 22.42	2.82:1	Gingiva (38.10%)
Melanoma	22 (9.13%)	56.18 ± 16.39	1.44:1	Gingiva (45.45%)
Physiologic pigmentation	5 (2.07%)	57.00 ± 9.27	4:1	Labial/buccal mucosa (50.00%) and gingiva (50.00%)
Smoker's melanosis	5 (2.07%)	41.00 ± 16.73	4:1	Gingiva (100.00%)
Drug-induced mucosal pigmentation	2 (0.84%)	24.50 ± 23.34	100:1	Labial/buccal mucosa (50.00%) and palate (50.00%)
Total	241 (100%)	38.74 ± 20.96	2.49:1	Gingiva (29.88%)

Abbreviation: SD, standard deviation.

## Discussion

Pigmented lesions can be physiologic and pathologic condition; some of them is associated with esthetics concern, 7 while some of them can be dangerous lesion. 8 The pigmented oral lesions in the present study constituted 0.51% of all biopsy cases from Thailand during the 20-year study period that is comparable to 0.07% by Ferreira et al, 9 0.83% by Buchner et al, 4 0.90% by Buchner et al, 10 1.34% by Tavares et al, 3 5.70% by De Giorgi et al, 11 but lower than 13.50% by Amir et al, 5 and30.30% by Hassona et al. 1 The explanation for the high prevalence of pigmented oral lesions in the study by Hassona et al 1 is accounted for by the fact that their study is a clinical study and the four most common pigmented lesions are racial pigmentation, smokers' melanosis, amalgam tattoo, and melanocytic macule. These types of lesions are usually diagnosed in the clinic and are not submitted for histopathological examination. Likewise, the study by Amir et al 5 focused exclusively on physiologic pigmentation in Israeli children, thus demonstrating a relatively high prevalence. Mean age of the patients with pigmented oral lesions in the present study (38.74 years) is comparable to 32.00 years by Buchner et al, 10 36.60 years by Ferreira et al, 9 39.00 years by De Giorgi et al, 11 41.3 years by Hassona et al, 1 43.70 years by Buchner et al, 4 and 45.00 years by Tavares et al. 3 The majority of pigmented oral lesions in the present study were encountered in females (71.37%) that are in accordance with the study by De Giorgi et al, 11 Buchner et al, 10 Ferreira et al, 9 Tavares et al, 3 and Hassona et al. 1 Certain pathologic entities in which the number of samples was high enough to draw a meaningful conclusion such as nevus, melanotic macule, and amalgam tattoo elicited a two- to threefold F:M ratio.

The three most common pigmented oral lesions in the present study were nevus (39.83%), followed by melanotic macule (28.63%) and amalgam tattoo (17.43%) that are similar to previous stories 3 4 12 despite the difference in order and slight pathological entity difference.

Nevi can present as a macule or a papule with pigmentation. It represents the most frequent pigmented oral lesion in the present study accounting for 0.20% of all accessioned cases and 39.83% of all pigmented lesions that are comparable to 0.06% of all the accessioned cases by Ferreira et al 9 and 0.10% of all the accessioned cases by Buchner et al, 4 but higher than 9.90% by De Giorgi et al, 11 11.80% of by Buchner et al, 10 20.50% of all pigmented lesions by Tavares et al. 3 Mean age of the patients with nevi in the present study was 29.80 years that is comparable to 30.50 years by Buchner et al, 4 32.00 years by Buchner et al, 10 and 36.60 years by Ferreira et al. 9 Nevi afflicted females more than males as in the previous studies. 3 4 9 10 13 The predilection site for nevi in the present study was labial/buccal mucosa (41.7%) followed by gingiva and palate (17.70% each). This is different from most previous studies 4 9 10 13 which consistently revealed that palate was the predilection site for nevi. This difference may be attributed to the racial difference since most of the patients from previous studies were Caucasians or Blacks, while all the patients in the present study were Asians. Intramucosal nevus constituted the most common histologic subtype of nevus in the present study (66.67%) that is in accordance with previous studies. 3 4 9 10 13 The second most frequent histologic subtype of nevus in the present study was compound nevus (25.03%) followed by blue nevus (8.33%). No junctional nevi were found in the study as reported by Buchner et al that this entity is very rare in oral cavity. 10 Previous studies 3 9 10 13 reported blue nevus was the second most common histologic subtype of nevus followed by compound nevus, while the study by Buchner et al 4 showed an equal distribution between compound nevus and blue nevus.

Melanotic macule is a small well-circumscribed brown-to-black macule that occurs on the lip and oral mucosa. Lip lesion is referred to as labial melanotic macule, while those on other parts of the oral mucosa are referred to as oral melanotic macule. 4 14 Melanotic macule in the present study accounted for 28.63% of all pigmented oral lesions that is in accordance with 22.90% by Tavares et al, 3 but lower than 58.30% by Pennacchiotti et al, 12 and 86.10% by Buchner et al, 4 but higher than 5.70% by Hassona et al, 1 and 13.30% by De Giorgi et al. 11 Mean age of the patients with melanotic macule in the present study was 40.83 years that is comparable to 37.00 years by De Giorgi et al, 11 43.70 years by Buchner et al, 4 but higher than 8.90 years by Pennacchiotti et al. 12 The reason why mean age of melanotic macule patients in the study by Pennacchiotti et al 12 is low is because that study was performed on Latin American children. Mean age of the patients with labial melanotic macule in the present study was 28.19 years, while that of the patients with oral melanotic macule in the present study was 44.74 years. It is noteworthy that the mean age of the oral melanotic macule patients is higher than that of the labial melanotic macule patients. The plausible explanation for this is that lip lesion is more noticeable by both the patient and the clinician than its oral counterpart. Both labial melanotic macule and oral melanotic macule elicited a female predominance that is in accordance with previous studies. 1 3 4 11 The present study along with previous studies 3 4 12 15 reported that oral melanotic macule outnumbered labial melanotic macule except the study by Hassona et al 1 that reported the lower vermillion as the most predilection site (82.00%) and the study by De Giorgi et al 11 that showed an equal distribution between oral melanotic macule and labial melanotic macule.

Amalgam tattoo is a dark gray or blue pigmented macular lesion from traumatic implantation of amalgam into the soft tissues. It was the third most common lesion in the present study comprising 0.09% of all accessioned cases and 17.43% of pigmented oral lesions thar are comparable to 0.06% of all accessioned cases by Tavares et al. 3 Mean age of the patients with amalgam tattoo in the present was 45.02 years that is in accordance with the study by Buchner and Hansen (43.10 years). 16 Amalgam tattoo showed the female predominance as in previous studies 12 16 17 and was preferentially encountered at gingiva (38.10%) and alveolar mucosa (33.33%) as in previous studies, 3 16 but different from the study in Brazil that revealed buccal mucosa as the site of predilection. 17

Melanoma is a cancer of pigment-producing cells, melanocytes. Clinical appearance of melanoma can be varied from patch, nodule, or even macule in the oral cavity. 8 The clinical evaluation of the symmetry, border, color change, and size of oral pigmented lesion is important for clinical diagnosis of melanoma. 18 In this study, it was the fourth most common lesion in the present study accounting for 0.05% of all accessioned cases and 9.13% of all pigmented oral lesions. The number of melanoma cases in this study is quite high. This is accounted for by the fact that this study is a multicenter study from 5 out of 7 major oral pathology biopsy service centers distributed in every corner of Thailand over a 20-year period so we get referral cases especially difficult and malignant cases. Pour et al found 17 melanoma cases in the face area out of 31,181 biopsy or autopsy cases in the northern part of Thailand during the 10-year study period. 19 Dhanuthai et al reported 10 melanoma cases from the oral cavity during 2005 to 2014 from Thailand. 20 Mean age of the patients with melanoma in the present study was 56.18 years that is in accordance with previous studies, 3 4 21 22 but lower than 66.7 years by Smith et al. 23 Some studies 3 23 24 including the present one showed a slight female predominance, while others revealed the opposite. 4 21 22 The present study revealed that gingiva was the most common site for melanoma (45.45%) followed by palate (31.82%) that is consistent with previous studies 4 17 19 that listed palate as the most common site for melanoma followed by gingiva 25 despite in reverse order or an equal distribution between palate and gingiva. 21

## Conclusions

The most common pigmented oral lesion in the present study is oral nevus followed by melanotic macule and amalgam tattoo. Demographic data of the patients in the present study are in accordance with previous studies with only minor differences. Pigmented lesions of the oral cavity may constitute a small portion of the oral pathology biopsies but accurate pathological diagnosis is important since there is an overlap in clinical appearance of benign pigmented lesions and melanoma. Therefore, when in doubt, biopsy must be done to reveal the accurate diagnosis of these group of lesions.
